# Supplementary material for: A comparative analysis of COVID-19 physical distancing policies in South Africa and Uganda
Source: PLOS Glob Public Health. 2024 Jul 3;4(7):e0003170. doi: 10.1371/journal.pgph.0003170 (PMC11221695; doi:10.1371/journal.pgph.0003170)
Supplement: S1 Appendix — (DOCX) [file pgph.0003170.s001.docx]

**Standardized Data Collection Form**

**Country or jurisdiction ________________________**

**WHO Region ________________________________ (African Region, Region of the Americas, South-East Asia Region, European Region, Eastern Mediterranean Region, Western Pacific Region) [**[**http://origin.who.int/about/regions/en/**](http://origin.who.int/about/regions/en/)**]**

**Data collector(s) ______________________________________________________________________**

**Use sources shown in the “sources” column, where marked, or if you use a different source, make a note of it. If blank, these will require varied sources depending on the country or jurisdiction. Write in all sources used and note what information is found from each source.**

**Part 1: Timeline**

| **Events** | **Findings** | **Comments** | **Sources** |
| --- | --- | --- | --- |
| Date WHO declared a Public Health Emergency of International Concern | January 30, 2020 |  | Rolling updates on coronavirus disease (COVID-19). WHO. (Updated regularly). <https://www.who.int/emergencies/diseases/novel-coronavirus-2019/events-as-they-happen> |
| Date pandemic declared | March 11, 2020 |  | WHO Timeline - COVID-19. WHO  (Updated April 27, 2020) <https://www.who.int/news-room/detail/27-04-2020-who-timeline---covid-19> |
| Date of country’s/jurisdiction’s first case |  |  | WHO Coronavirus Disease (COVID-19) Dashboard  (Updated regularly)  <https://covid19.who.int/> |
| Date of country’s/jurisdiction’s 100 cases |  |  | WHO Coronavirus Disease (COVID-19) Dashboard  (Updated regularly)  <https://covid19.who.int/> |
| Global Health Security Overall Rank # and category |  |  | Global Health Security Index  <https://www.ghsindex.org/> |
| Global Health Security Epidemic Preparedness Rank # and category |  |  | Global Health Security Index  <https://www.ghsindex.org/> |
| Other: |  |  |  |
| Other: |  |  |  |

**Part 2: Country characteristics – geographic, environmental, social, economic, demographic and health**

| **GEOGRAPHIC** and **ENVIRONMENTAL** | | | |
| --- | --- | --- | --- |
| **Category** | **Findings** | **Comments** | **Sources** |
| Population of country / jurisdiction |  |  | Countries in the world by population (2020). Worldometer. (n.d.) <https://www.worldometers.info/world-population/population-by-country/> |
| Land area of country / jurisdiction (km^2^) |  |  | Countries in the world by population (2020). Worldometer. (n.d.) <https://www.worldometers.info/world-population/population-by-country/> |
| Agricultural land (%) |  |  | Central Intelligence Agency World Factbook. <https://www.cia.gov/library/publications/the-world-factbook/> |
| Population distribution- description |  |  | Central Intelligence Agency World Factbook. <https://www.cia.gov/library/publications/the-world-factbook/> |
| Population density of country / jurisdiction (#/ km^2^) |  |  | Countries in the world by population (2020). Worldometer. (n.d.) <https://www.worldometers.info/world-population/population-by-country/> |
| Urban population (%) |  |  | Countries in the world by population (2020). Worldometer. (n.d.) <https://www.worldometers.info/world-population/population-by-country/> |
| Geography – brief description |  |  | Central Intelligence Agency World Factbook <https://www.cia.gov/library/publications/the-world-factbook/> |
| PM2.5 air pollution, mean annual exposure, 2017 (micrograms per cubic meter) |  |  | The World Bank - PM2.5 -  <https://data.worldbank.org/indicator/EN.ATM.PM25.MC.M3?view=chart>  PM2.5 For Canadian Provinces (2016): <https://www.canada.ca/content/dam/eccc/documents/pdf/cesindicators/air-quality/air-quality-en.pdf> |
| PM2.5 air pollution, population exposed to levels exceeding WHO guideline value, 2017 (% of total) |  |  | The World Bank - PM2.5 -  <https://data.worldbank.org/indicator/EN.ATM.PM25.MC.ZS?view=chart> |
| **SOCIAL** | | | |
| **Category** | **Findings** | **Comments** | **Sources** |
| Social connectedness Social infrastructure - Individualism vs. collectivism vs. tribalism vs. solidarity |  |  |  |
| Ethnic groups and % |  |  | Central Intelligence Agency World Factbook <https://www.cia.gov/library/publications/the-world-factbook/> |
| Number of different ethnic groups (based on above) |  |  | Add above |
| Languages spoken and % |  |  | Central Intelligence Agency World Factbook <https://www.cia.gov/library/publications/the-world-factbook/> |
| Number of different languages spoken(based on above) |  |  | Add above |
| Religions and % |  |  | Central Intelligence Agency World Factbook <https://www.cia.gov/library/publications/the-world-factbook/> |
| Number of different religions (based on above) |  |  | Add above |
| Trust in government |  |  | Our World In Numbers (Updated 2018)  <https://ourworldindata.org/trust> |
| Mobile cellular subscriptions (per 100 people) |  |  | World Bank open data found at: <https://data.worldbank.org/> |
| Individuals using the Internet (% of population) |  |  | World Bank open data - found at: <https://data.worldbank.org/> |
| Other: |  |  |  |
| **ECONOMIC** | | | |
| **Category** | **Findings** | **Comments** | **Sources** |
| Economic framework – Index of economic freedom (composite of rule of law, government size, regulatory efficiency, and open markets) **Categorical** - Free, Mostly free, Moderately free, Mostly unfree, Repressed, Not ranked) **and rank** |  |  | 2020 Index of Economic Freedom  <https://www.heritage.org/index/ranking> |
| World Bank classification – Low, lower middle, upper middle, high |  |  | World Bank: World by Income & Region (2020)  <https://datatopics.worldbank.org/world-development-indicators/the-world-by-income-and-region.html> |
| GINI Index |  |  | Central Intelligence Agency World Factbook - Gini  <https://www.cia.gov/library/publications/the-world-factbook/rankorder/2172rank.html> |
| GDP per capita, PPP, (current international $) |  |  | World Bank open data - GDP - found at: [https://data.worldbank.org/indicator/NY.GNP.PCAP.PP.CD?view=chart/](https://data.worldbank.org/) |
| GNI per capita, PPP (current international $) |  |  | World Bank open data - GNI - found at: <https://data.worldbank.org/indicator/NY.GNP.PCAP.PP.CD?view=chart> |
| Current health expenditure (%) |  |  | Central Intelligence Agency World Factbook <https://www.cia.gov/library/publications/the-world-factbook/> |
| Vulnerable employment, total (% of total employment) |  |  | World Bank  <https://data.worldbank.org/indicator/SL.EMP.VULN.ZS> |
| Vulnerable employment, female (% of female employment) |  |  | World Bank  <https://data.worldbank.org/indicator/SL.EMP.VULN.FE.ZS> |
| Vulnerable employment, male (% of male employment) |  |  | World Bank  <https://data.worldbank.org/indicator/SL.EMP.VULN.MA.ZS> |
| **DEMOGRAPHIC and HEALTH** | | | |
| **Category** | **Findings** | **Comments** | **Sources** |
| Population ages 0-14 (total and %)  % |  |  | World Bank open data found at: <https://data.worldbank.org/indicator/SP.POP.0014.TO>  <https://data.worldbank.org/indicator/SP.POP.0014.TO.ZS> |
| Population ages 15-64 (total and %)  % |  |  | World Bank open data found at: <https://data.worldbank.org/indicator/SP.POP.1564.TO>  <https://data.worldbank.org/indicator/SP.POP.1564.TO.ZS> |
| Population ages 65 and above (total and %) |  |  | World Bank open data found at: <https://data.worldbank.org/indicator/SP.POP.65UP.TO>  <https://data.worldbank.org/indicator/SP.POP.65UP.TO> |
| Population ages 0-14, female (total and %) |  |  | World Bank open data found at: <https://data.worldbank.org/indicator/SP.POP.0014.FE.IN> |
| Population ages 0-14, male (total and %) |  |  | World Bank open data found at: <https://data.worldbank.org/indicator/SP.POP.0014.MA.IN> |
| Population ages 15-64, female (total and %) |  |  | World Bank open data found at: <https://data.worldbank.org/indicator/SP.POP.1564.FE.IN>[/](https://data.worldbank.org/) |
| Population ages 15-64, male (total and %) |  |  | World Bank open data found at: <https://data.worldbank.org/indicator/SP.POP.1564.MA.IN> |
| Population ages 65 and above, female (total and %) |  |  | World Bank open data found at: <https://data.worldbank.org/indicator/SP.POP.65UP.FE.IN> |
| Population ages 65 and above, male (total and %) |  |  | World Bank open data found at: <https://data.worldbank.org/indicator/SP.POP.65UP.MA.IN> |
| Life expectancy at birth 2017 (yrs) |  |  | World Bank: Life Expectancy <https://data.worldbank.org/indicator/sp.dyn.le00.in> |
| Current tobacco smoking, adults aged 15+, total (%) |  |  | World Bank: Smoking  <https://data.worldbank.org/indicator/SH.PRV.SMOK> |
| Current tobacco smoking, adults aged 15+, males (%) |  |  | World Bank: Smoking  <https://data.worldbank.org/indicator/SH.PRV.SMOK.MA> |
| Current tobacco smoking, adults aged 15+, females (%) |  |  | World Bank: Smoking  <https://data.worldbank.org/indicator/SH.PRV.SMOK.FE> |
| Raised blood pressure, adults aged 18+, total (%) |  |  | WHO noncommunicable diseases by country -  <https://www.who.int/nmh/countries/en/> |
| Raised blood pressure, adults aged 18+, males (%) |  |  | WHO noncommunicable diseases by country -  <https://www.who.int/nmh/countries/en/> |
| Raised blood pressure, adults aged 18+, females (%) |  |  | WHO noncommunicable diseases by country -  <https://www.who.int/nmh/countries/en/> |
| (Diabetes) Raised blood glucose, adults aged 18+, total (%) |  |  | WHO noncommunicable diseases by country -  <https://www.who.int/nmh/countries/en/> |
| (Diabetes) Raised blood glucose, adults aged 18+, males (%) |  |  | WHO noncommunicable diseases by country -  <https://www.who.int/nmh/countries/en/> |
| (Diabetes) Raised blood glucose, adults aged 18+, females (%) |  |  | WHO noncommunicable diseases by country -  <https://www.who.int/nmh/countries/en/> |
| Obesity, adults aged 18+, total (%) |  |  | WHO NCD by country  <https://www.who.int/nmh/countries/en/> |
| Obesity, adults aged 18+, males (%) |  |  | WHO NCD by country  <https://www.who.int/nmh/countries/en/> |
| Obesity, adults aged 18+, females (%) |  |  | WHO NCD by country  <https://www.who.int/nmh/countries/en/> |
| Proportional mortality from cardiovascular diseases (%) |  |  | WHO NCD by country  <https://www.who.int/nmh/countries/en/> |
| Proportional mortality from cancers (%) |  |  | WHO NCD by country  <https://www.who.int/nmh/countries/en/> |
| Proportional mortality from chronic respiratory diseases (%) |  |  | WHO NCD by country  <https://www.who.int/nmh/countries/en/> |
| Proportional mortality from diabetes (%) |  |  | WHO NCD by country  <https://www.who.int/nmh/countries/en/> |
| Prevalence of HIV in adult population (%) |  |  | Central Intelligence Agency World Factbook (2018 est) <https://www.cia.gov/library/publications/the-world-factbook/> |
| BCG  Immunization coverage estimates by country (%) |  |  | WHO - BCG Immunization coverage  <https://apps.who.int/gho/data/node.main.A830?lang=en> |
| Homelessness (%) |  |  | OECD  <https://www.oecd.org/els/family/HC3-1-Homeless-population.pdf> |
| International migrant stock (% of population) |  |  | World Bank <https://data.worldbank.org/indicator/SM.POP.TOTL.ZS> |
| Prevalence of undernourishment (% of population) |  |  | World Bank  <https://data.worldbank.org/indicator/SN.ITK.DEFC.ZS?view=chart> |
| Adult literacy rate 2015 (%) |  |  | UNESCO Institute for Statistics <http://uis.unesco.org/#slideoutsearch> |
| Literacy rate, adult male (% of males 15 and above) |  |  | UNESCO Institute for Statistics <http://data.uis.unesco.org/index.aspx?queryid=121> |
| Literacy rate, adult female (% of females 15 and above) |  |  | UNESCO Institute for Statistics <http://data.uis.unesco.org/index.aspx?queryid=121> |
| Primary school net enrolment ratio 2015 (%) |  |  | World Bank  <https://data.worldbank.org/indicator/SE.PRM.NENR> |

**Political and health system**

| **POLITICAL SYSTEMS** | | | |
| --- | --- | --- | --- |
| **Category** | **Findings** | **Comments** | **Sources** |
| Type of government – democratic vs. authoritarian (monarchy, dictatorship, single-party) |  |  | Forms of government. Lumen. (n.d.) <https://courses.lumenlearning.com/boundless-politicalscience/chapter/forms-of-government>/ |
| Type of government - other description |  |  | Central Intelligence Agency World Factbook  <https://www.cia.gov/library/publications/the-world-factbook/fields/299.html> |
| Date since current government in place |  |  | Central Intelligence Agency World Factbook  <https://www.cia.gov/library/publications/the-world-factbook/fields/313.html#BC> |
| Election Term Length |  |  | <https://en.wikipedia.org/wiki/List_of_next_general_elections> |
| Fragile States Index - score (out of 120) [higher is worse] and FSI rank (out of 178 countries) [higher is better] |  |  | Fragile States Index - Country dashboard.  <https://fragilestatesindex.org/country-data/> |
| Global Freedom Score  Number and status |  |  | <https://freedomhouse.org/countries/freedom-world/scores>  Global freedom score category and number |
| Internet Freedom Score Number and status |  |  | <https://freedomhouse.org/countries/freedom-net/scores> |
| Freedom of the press score and rank (0-60: high \| 61-120: medium \| 121-180: low) |  |  | <https://rsf.org/en/ranking> |
| Name of governing party in power and leaning |  |  | Political spectrum. Wikipedia. (Updated April 20, 2020) <https://en.wikipedia.org/wiki/Political_spectrum> Check how the governing party describes itself (conservative, liberal, progressive, etc), then determine how it fits into the range of political ideologies in the jurisdiction. For example, the BC Liberal Party is the conservative party in that jurisdiction. |
| Multilevel government (federal vs. unitary)  Sub-questions:   1. Is there a constitutional division of power for health? 2. Level of decentralization |  |  | Degree of centralization re: policy authority for health is the most relevant measure, but useful to note if a country has a federal constitution (distinct constitutional power for different levels of government). A quick search of the country name + federalism can determine constitutional structure. See also brief op-eds by in-country experts on the role of federalism in COVID response in a number of federal countries: <http://www.forumfed.org/collection/federalism-and-covid-country-perspectives/page/2/> |
| For democratic governments, party discipline (Yes, No) |  |  |  |
| Policy authority for health and level of involvement from levels of government (decision making power, funding decisions, consider health regions) |  |  | International Health Care Systems Profiles (Commonwealth Countries)  <https://international.commonwealthfund.org/countries/> |
| Policy authority for education |  |  | World Education News  <https://wenr.wes.org/category/education-system-profiles> |
| Policy authority for international travel |  |  | International Air Transportation Authority  <https://www.iata.org/en/programs/safety/health/diseases/government-measures-related-to-coronavirus/> |
| Policy authority for emergency management |  |  |  |
| Act(s) determining policy authority for emergency management |  |  |  |
| Interest groups – any prominent groups involved or pushing for certain style of policies |  |  | Suggest labour unions (e.g. CLC in Canada), chambers of commerce, and individual large companies/industries (example for Canada, check iPolitics’ Lobby Wrap <https://ipolitics.ca/2020/05/18/lobby-wrap-air-canada-chl-lobbying-for-financial-aid/>)  Religious groups |
| Ideas – beliefs or values (public opinion polls, protests) |  |  | Possible to measure trust in government and confidence in government response to pandemic using public opinion data? Vox Pop is fielding a rolling study for Canada: <https://voxpoplabs.com/covid-19/> |
| External factors |  |  |  |
| **HEALTH SYSTEMS** | | | |
| Health financing – public, private, mixed |  |  | International Health Care Systems Profiles (Commonwealth Countries)  <https://international.commonwealthfund.org/countries/> |
| National Centre for Disease Control |  |  | Where We Work - CDC  <https://www.cdc.gov/globalhealth/countries/default.htm> |
| Central health authority |  |  | International Health Care Systems Profiles (Commonwealth Countries)  <https://international.commonwealthfund.org/countries/> |
| Number of health districts |  |  | International Health Care Systems Profiles (Commonwealth Countries)  <https://international.commonwealthfund.org/countries/> |
| Physician density (physician/1,000 pop) |  |  | Central Intelligence Agency World Factboo[k](https://www.cia.gov/library/publications/the-world-factbook/) <https://www.cia.gov/library/publications/the-world-factbook/> |
| Hospital bed density (beds/1,000 pop) |  |  | Central Intelligence Agency World Factbook <https://www.cia.gov/library/publications/the-world-factbook/> |
| National stockpile PPE |  |  | WHO - countries who have received WHO PPE  <https://www.who.int/news-room/detail/03-03-2020-shortage-of-personal-protective-equipment-endangering-health-workers-worldwide>  *Scroll to the bottom (note to editor) for a list of countries who received PPE from WHO* |
| Emergency preparedness planning – experiences with pandemics in past 25 years |  |  | <https://www.ghsindex.org/> |
| Pandemic response plan - Y/N (date last updated) |  |  |  |
| Hazard Risk and Vulnerability Assessment (or similar plan) - Y/N and date last updated |  |  |  |
| Laboratory landscape – public health, private labs |  |  |  |
| COVID-19 testing policy  Availability of testing |  |  | Our World in Data  <https://ourworldindata.org/grapher/covid-19-testing-policy?year=2020-05-08> |
| COVID-19 test used and where developed |  |  |  |
| COVID-19 antibody test used and where developed |  |  |  |
| Surveillance systems and data collected |  |  |  |
| What is known about COVID outbreaks in the country with regards to local outbreaks (long-term care, hospital, occupational, prisons, etc.) High-level overview (see epidemiology section for further details) |  |  |  |

| **Policies** | | | | | |
| --- | --- | --- | --- | --- | --- |
| **Policy** | **Findings - yes/no or categorical** | **Date started** | **Date ended** | **High-level summary of policy and modifications made (with dates)** | **Sources** |
| COVID-19 primary spokesperson(s) |  |  |  |  |  |
| Declared planned approach to pandemic – **Categorical** - containment, mitigation, herd immunity, re-opening, other |  |  |  |  |  |
| Change(s) in declared approach(es), dates and reasons provided |  |  |  |  |  |
| Declaration of state of emergency - Y/N |  |  |  |  |  |
| **Distancing measures** | | | | | |
| Physical distancing - Y/N and recommendation (distance) |  |  |  |  |  |
| Ban on group sizes for gatherings - Y/N (Description - size number and exception notes, e.g., religious gatherings, sporting events, bars/restaurants) |  |  |  |  |  |
| School closures, Daycares -Y/N |  |  |  |  |  |
| School closures, Primary and secondary - Y/N |  |  |  |  |  |
| School closures, Universities - Y/N |  |  |  |  |  |
| Closing non-essential services - Y/N  (with description) |  |  |  |  |  |
| Closing restaurants |  |  |  |  |  |
| Suspended elective medical/dental procedures |  |  |  |  |  |
| Separation of cases or suspected cases within institutions or in separate institutions - Y/N |  |  |  |  |  |
| Health workers allowed to only work at one site (hospital) - Y/N |  |  |  |  |  |
| Health workers allowed to only work at one site (senior care facilities) - Y/N |  |  |  |  |  |
| Isolation for vulnerable populations (elderly, immunocompromised) - Y/N |  |  |  |  |  |
| Quarantine orders after travel - Y/N |  |  |  |  | International Pandemic Preparedness  <https://pandemic.internationalsos.com/2019-ncov/ncov-travel-restrictions-flight-operations-and-screening> |
| Quarantine orders for cases - Y/N |  |  |  |  |  |
| Quarantine orders for contacts - Y/N |  |  |  |  |  |
| Recommended self-isolation after travel - Y/N |  |  |  |  |  |
| Recommended self-isolation for cases- Y/N |  |  |  |  |  |
| Recommended self-isolation with symptoms - Y/N |  |  |  |  |  |
| Recommended self-isolation for contacts - Y/N |  |  |  |  |  |
| Work-from-home / remote work - Y/N |  |  |  |  |  |
| Quarantine for “at risk” or “priority” neighborhoods - Y/N |  |  |  |  |  |
| Lockdown - Y/N |  |  |  |  |  |
| Other: |  |  |  |  |  |
| Other: |  |  |  |  |  |
| **Identification, containment and mitigation measures** | | | | | |
| International bans for non-essential travel - Y/N |  |  |  |  | International Air Transport Authority  <https://www.iata.org/en/programs/safety/health/diseases/government-measures-related-to-coronavirus/>  European Union  <https://ec.europa.eu/info/live-work-travel-eu/health/coronavirus-response/travel-and-transportation-during-coronavirus-pandemic_en> |
| Closing public transportation - Y/N |  |  |  |  |  |
| Screening at airports / borders - Y/N |  |  |  |  | International Air Transport Authority  <https://www.iata.org/en/programs/safety/health/diseases/government-measures-related-to-coronavirus/>  European Union  <https://ec.europa.eu/info/live-work-travel-eu/health/coronavirus-response/travel-and-transportation-during-coronavirus-pandemic_en> |
| Contact tracing - Y/N |  |  |  |  |  |
| Assessment centres - Y/N |  |  |  |  |  |
| Drive-through testing sites - Y/N |  |  |  |  |  |
| Mass fever screening in public transportation - Y/N |  |  |  |  |  |
| RECOMMENDED use of masks/PPE for public - Y/N |  |  |  |  |  |
| REQUIRED use of masks/PPE for public - Y/N |  |  |  |  |  |
| Public decontamination transit - Y/N |  |  |  |  |  |
| Public decontamination streets - Y/N |  |  |  |  |  |
| **Social and economic policies - support adherence to other policies** | | | | | |
| Economic relief policies for individuals / families - Y/N |  |  |  |  |  |
| Housing economic relief (eviction freeze, rent/mortgage deferral) |  |  |  |  |  |
| Economic relief policies for businesses - Y/N |  |  |  |  |  |
| Anti-price gouging - Y/N |  |  |  |  |  |
| Anti-hoarding - Y/N |  |  |  |  |  |
| Audio/video telehealth |  |  |  |  |  |
| Telehealth access to prescription medication |  |  |  |  |  |

**Epidemiology of COVID-19**

| **Date** | **Category** | **Findings** | **Comments** | **Sources** |
| --- | --- | --- | --- | --- |
|  | Total number of cases |  |  | Worldometer  <https://www.worldometers.info/coronavirus/> |
|  | Total number of deaths |  |  | Worldometer  <https://www.worldometers.info/coronavirus/> |
|  | Total number tested |  |  | Worldometer  <https://www.worldometers.info/coronavirus/> |
|  | Total number recovered |  |  | Worldometer  <https://www.worldometers.info/coronavirus/> |
|  | Total number of cases in hospital |  |  |  |
|  | Total number in ICU |  |  |  |
|  | Total number on ventilators |  |  |  |
|  | Total in long term / nursing / retirement / older adult congregate living care |  |  |  |
|  | Total in prisons |  |  | Correctional Services Canada  <https://www.csc-scc.gc.ca/001/006/001006-1014-en.shtml>  US Bureau of Prisons  <https://www.bop.gov/coronavirus/index.jsp>  European Prison  <https://www.prison-insider.com/en/articles/europe-coronavirus-la-fievre-des-prisons> |
|  | Nosocomial infections / number of outbreaks in hospitals |  |  |  |
|  | Number of cases linked to non-health occupational exposure (and description of occupation) |  |  |  |
|  | Number of cases linked to health occupational exposure (and description of occupation) |  |  |  |
|  | Number of cases / 1M |  |  | Worldometer  <https://www.worldometers.info/coronavirus/> |
|  | Number of deaths / 1M |  |  | Worldometer  <https://www.worldometers.info/coronavirus/> |
|  | Numbers tested / 1M |  |  | Worldometer  <https://www.worldometers.info/coronavirus/> |
|  | % of cases testing positive |  |  | DOMO  <https://www.domo.com/covid19/testing-and-treatment#testing> |
|  | Total number of cases - male |  |  | Link to sources of data used by Worldometer by clicking on the country name. The original sources is listed under “updates” |
|  | Total number of cases - female |  |  | Link to sources of data used by Worldometer by clicking on the country name. The original sources is listed under “updates” |
|  | Total number of deaths - male |  |  | Link to sources of data used by Worldometer by clicking on the country name. The original sources is listed under “updates” |
|  | Total number of deaths - female |  |  | Link to sources of data used by Worldometer by clicking on the country name. The original sources is listed under “updates” |
|  | Total number of tested - male |  |  | Link to sources of data used by Worldometer by clicking on the country name. The original sources is listed under “updates” |
|  | Total number of tested - female |  |  | Link to sources of data used by Worldometer by clicking on the country name. The original sources is listed under “updates” |
|  | Case definition for testing and changes (with dates) |  |  | Link to sources of data used by Worldometer by clicking on the country name. The original sources is listed under “updates” |
|  | Other: |  |  |  |
|  | Other: |  |  |  |
