# Supplementary material for: A comparative analysis of COVID-19 physical distancing policies in South Africa and Uganda
Source: PLOS Glob Public Health. 2024 Jul 3;4(7):e0003170. doi: 10.1371/journal.pgph.0003170 (PMC11221695; doi:10.1371/journal.pgph.0003170)
Supplement: S2 Appendix — (DOCX) [file pgph.0003170.s002.docx]

Appendix 2: Interview Guide

**Semi-structured key informant interview guide (30-45 minutes)**

Participant ID__________________________________ Date_____________________________

Location________________________________________ Researcher________________________

Start time_______________ End time_______________ Date of informed consent ____________

Review and obtain informed consent - see informed consent form. Please note: (i) we are not part of any NGO or donor agencies; (ii) we will not offer you any kind of subsidy for interviewing you, (iii) we will keep your information anonymous, and will use only it for research, and (iv) you can stop me at any time. Are you agreed to be interviewed? (Yes) or (No). OK to record: Y / N

-**Thank you for agreeing to be part of this study. Have you had a chance to look over the interview questions that I sent you beforehand? I will first ask you some broad questions, and at the end, I will ask you some specific questions to help understand the context of this work. For this interview, “jurisdiction” means country or sub-national entity such as a province, state or territory. Do you have any questions before we start?**-

**Semi-structured interview questions**

**1) What is/was your role with regards to dealing with COVID-19?**

**Probes –** Were you involved in policy decisions to combat COVID-19?

Were you involved in developing or implementing policies?

Were you involved in public health actions, emergency management, or education decisions?

Were you involved in treating patients with COVID-19?

**2) From your perspective, can you describe the unfolding of COVID-19 in your jurisdiction?**

**Probes –** When was your first case discovered and how

Who was involved in decision making – government, public health

Who was the main spokesperson to the public?

How did things play out following the initial cases?

**3) From your perspective, what are the main sources of information used when making decisions about COVID-19 policy in your jurisdiction?**

**Probes**: President/Prime Minister, governor, mayor, international organizations, like the WHO? National scientific and/or medical bodies? Look to the experience of other jurisdictions? Consider the public response to different actions? Were decisions led by experts or political figures? Physical distancing policies and their effects on the epidemiology of COVID-19: A multi-national comparative study

**4) a)What was the overarching plan for the pandemic in your jurisdiction before WHO declared a Public Health Emergency of International Concern on January 30, 2020?**

**b) From January 30 and March 10 before WHO declared a pandemic?**

**c) After WHO declared a pandemic on March 10?**

**d) Did the decision about the overarching plan change over time, and if so, what influenced the change?**

**Probes** - Plan – containment, mitigation, herd immunity, other, no announced plan

Who decided and what was their rationale for this decision?

**5) Do you believe the plan (Covid-19 risk management policy measures such as lockdown, flight closure, quarantine, social distancing, disinfection, remote work, distance learning, etc) was successful? Why or why not? How do you measure success?**

**Probes -** Numbers tested, number of cases, numbers hospitalized, numbers dead, numbers recovered, other

**6) Which Covid-19 risk management policy measures worked well about this plan? What factors contributed to things that worked well about this plan?**

**Probes -** Consistency in messaging, communication, early action, public engagement

**7) Looking back, what would you suggest could have been done for better Covid-19 risk management in your country/jurisdiciton, why and how?**

**Probes -** Early testing, lockdown, flight bans, social distancing, better communication, different strategy

**8) (Possibly review questions surrounding specific policies that were unclear from earlier research)** Physical distancing policies and their effects on the epidemiology of COVID-19: A multi-national comparative study

**9) Who else would be good to speak with to get a clear understanding of policies and epidemiology of COVID-19 in your jurisdiction? Can you provide contact information for this/these individual(s)? Or, are there documents which may be relevant to understanding events surrounding the pandemic which I could access?**

**Probes –** Policymakers, researchers, healthcare professionals, public health officials, emergency managers and/or other stakeholders, surveillance data with breakdown of numbers nationally, by state and by municipality

**10) Is there any other information you feel I have left out which you would like to tell me regarding the response to COVID-19 in your jurisdiction?**

**- Before ending this interview, I need to gather some personal data for contextual factors -**

**Demographic information**

Age__________ Gender: F / M / Other Country or subnational jurisdiction_____________

Current professional designation______________ Length of time at current position __________

Other professional designation(s)_____________________

**Thank you for your time. Is it ok to contact you again if I need any clarifications or have other questions? Thanks again!** OK to contact for further interview: Y / N
