# Supplementary material for: A comparative analysis of COVID-19 physical distancing policies in South Africa and Uganda
Source: PLOS Glob Public Health. 2024 Jul 3;4(7):e0003170. doi: 10.1371/journal.pgph.0003170 (PMC11221695; doi:10.1371/journal.pgph.0003170)
Supplement: Attachment — Submitted filename: Response letter to reviewers.docx [file pgph.0003170.s004.docx]

Dear reviewers,

Thank you for your constructive feedback to strengthen our paper. We have addressed your comments below:

**Reviewer #1: Thank you for an excellent research paper about the comparative CoViD response in South Africa and Uganda.
while I found the paper well researched and thorough, I feel that there are some areas where you could supplement the material in the paper
1. Figures 1 and 2 show that there were three waves in South Africa and only two in Uganda.. as a matter of fact, in Uganda, the first wave (which was much smaller than any of the waves in South Africa) started only in Oct 2020 unlike in South Africa where the first wave was around August 2020. Both the countries implemented a nationwide lockdown in March. Uganda extended its lockdown to around June, while South Africa relaxed the same in May. Both had international travel bans in place. It is not clear from the various policy measures taken why the epidemiological pattern was so different.. and how Uganda escaped the wave in August 2020 or why the number of cases in Uganda in both waves was so much lower(as was the mortality)**

Thank you for reviewing the findings. We also find these findings intriguing! These case studies are meant to describe the events and policies in both countries; however, concrete conclusions about which specific policies were successful in one setting versus the other are not possible to make given the complexity of the policies, their overlap in timing, and the different contextual features in these settings. While Figures 1 and 2 capture the types of policies and their timing, these policies were not identical in definition or strictness in each jurisdiction. There may also have been a role in how proactive Uganda was with their policies – as we explain in the discussion section of the text, South Africa began implementing mitigation policies once their case numbers had already started rising, while Uganda they began implementing policies prior to detecting their first case.

**2.You have alluded to the population distribution of the country (age as well as rural/urban distribution) as one of the reasons for the relative success of Uganda viz a viz South Africa. Still the population density in Uganda is much higher.
Was there any data to support why the age/rural/urban mix was the reason why the disease spread more slowly.. for instance, were the cases more among the elderly or in the urban population**

Thank you for your comment. The contextual data were included in our research due to hypothetical influences on epidemiological trends. We selected these global indicators a priori thinking that population density, distribution, and age would play a role in COVID-19 transmission and outcomes. Given the nature of this study as a qualitative case study comparing 2 countries, these indicators represent country-level measures, and do not have the statistical power to provide a stratified analyses that could answer these questions. It is our hope that by comparing many different countries, we will be able to highlight general patterns in the characteristics of countries that had significant COVID-19 transmission. It is exactly these initial questions that led us to conduct this project – the realization that there are so many factors contributing to COVID-19 epidemiological trends that we would need nuanced understanding of the policies and the countries’ contexts.

**3. From the paper, we are not able to see whether there were any other factors such as implementational efficiency (state capacity), institutional arrangements, etc which resulted in Uganda doing a better job. You have described the governments in both states and also stated that the public supported the lockdown. Were there any features of state legitimacy in the months that followed that improved public compliance of social distancing? Were you able to elicit any of these from the qualitative interviews.
I feel these areas if elaborated with available data and interview response could strengthen the paper.**

Thank you. We agree that these are important considerations; however, the data are variable for different countries and difficult to compare. Based on comments from Reviewer 2, we added the data collection form as supplementary material to show the complete list of global indicators we collected. These indicators were selected a priori and limited to ones that were accessible and comparable globally. We added wording to the manuscript to explain this point further (Clean manuscript lines 193 -194).

Unfortunately, we did not have direct measures of state capacity or implementational efficiency when it came to compliance with COVID-19 policies. We included trust in national government as a proxy indicator to get a sense of whether people living in these countries were likely to follow COVID-19 policies; however, we understand that this indicator is limited. A separate paper by the COVID-19 Policies and Epidemiology Working Group addresses some of these factors and others as limitations in COVID-19 data. We have added language to highlight these limitations more clearly and provided a citation in the paper (Clean manuscript lines 810-811 and 814-816).

**Reviewer #2: The investigators set out to compare physical distancing measures implemented in South Africa and Uganda during the COVID-19 pandemic in terms of their approaches and outcomes.
Here are some comments and recommendations for the authors to consider:**
**1. To properly situate the discussion on the impact of the policies, an exploration of differences in public health interventions that also impact the outcome needs to be considered. For example, what were the policies on use of face masks and access to testing. Whilst masking policies may have impacted infection risk, access to testing impacts case finding.**

Thank you. We agree with the importance of these factors in an analysis for impact of policies. Given that we are including 2 countries in this paper and given the complexity of the number, timing, and stringency of policies, it is challenging to conduct a statistical analysis on how specific interventions impact outcomes. We have data on when masks were used, as well as policies on access to testing and contact tracing. However, implementation and public compliance with these policies remains unclear and it is difficult to assess their impact overall. We have included recommended and mandatory mask policies in the policy graphs for each country (Fig 1 and Fig 2). We have also included our data collection form as an appendix which shows the physical distancing and related policies that we set out to study a priori and provided a link to our website where we have shared our policy-relevant database summarizing the information collected for each country (Clean manuscript lines 189 and 244-247).

**2. Potential differences in completeness of reporting needs to be accounted for if you are going to make inferences about the impact of public health interventions during COVID. Were there differences in timeliness and completeness of reporting between South Africa and Uganda?**

Thank you. We agree these are important considerations. Both countries reported data publicly using televised press conferences, press releases, and online. A separate paper from the COVID-19 Policies and Epidemiology Working Group outlines the limitations of COVID-19 data. We have clarified this limitation in the manuscript and provide a citation (Clean manuscript lines 808-810 and 814-816).

**3. There is some justification in the text, but I was not entirely sure of the rationale for comparing SA and Uganda, especially considering their income differences.**

Thank you. The rationale for this comparison is stated in the background section of the manuscript. South Africa and Uganda were chosen based on how their early COVID-19 responses were perceived internationally. Uganda’s response was celebrated for its success even with their income classification. Logistically, both countries are English-speaking and were collecting and reporting on COVID-19 publicly on a regular basis. This study is also part of a larger study by the COVID-19 Policies and Epidemiology Working group, which explores COVID-19 responses across multiple countries. As part of the study, we wanted to ensure that there were African countries represented in our results (Clean manuscript lines 124-143).

**4. Overall, the manuscript is substantially longer than it needs to be. A lot of the material in the results can be displayed in tables instead.**

Thank you. We agree that data should be presented in a concise manner, therefore we have added Table 2: Contextual factors and Table 3: Population age and health characteristics to summarize data on other factors that may affect COVID-19 transmission. We have also shortened the accompanying text descriptions (Clean manuscript lines Table 2: 311-312 and Table 3: 392-393).

Introduction
**• Page 4 line 64-66: occupational exposure was an important factor particularly in the early days of the pandemic.**

Thank you. This factor was added to the text and cited (Clean manuscript line 66).

**• Page 6 line 120-123: please provide references.**

Thank you. References have been added (Clean manuscript lines 122-123).

**• Page 7 line 141-142: sentence beginning “contextual factors…” is redundant. Consider removing.**

Thank you. This sentence was removed.

**• Page 7 line 142-145: please clarify how you came to the conclusion on transparency in COVID-19 data collection and reporting.**

Thank you. We did not have a direct transparency indicator. In fact, this statement was about having publicly available COVID-19 data that were updated on a regular basis. The wording was updated accordingly (Clean manuscript lines 141-142).

Methods
**• Please include the data collection form as an appendix or supplementary material.**

Thank you. This material has been attached as S1 Appendix and referenced in the text (Clean manuscript line 189).

**• Please clarify if you sought (perhaps from the informants) unpublished memos or other documents that may have been relevant. Also applies to documents that may have been published in print or were otherwise unavailable on the internet.**

Thank you. Yes, we did seek documents that may be relevant to understanding events surrounding the pandemic as part of the interview process. The semi-structured interview guide has been added as S2 Appendix. We have clarified in the text (Clean manuscript lines 227 and 232-233).

No documents were gathered through this process that were not already publicly available (Clean manuscript lines 293 -295).

**• Please provide more details on your content analysis procedure. How did you manage codes or themes, for example? Details to enable the reproducibility of your results need to be provided.**

Thank you. We agree that it is important to provide details that allow for the results to be reproduced. Data were collected following a standardized data collection form. The information was descriptive, and data were triangulated for dates and policy descriptions. We used deductive content analysis guided by the data collection form. We have added these additional details to the text (Clean manuscript lines 237 and 242).

**• Please clarify if local ethics approval was obtained in SA and Uganda.**

Thank you. Ethics approval was obtained in Canada since the research was conducted from Canada and included key informant interviews with individuals from South Africa and Uganda. We have clarified in the text (Clean manuscript lines 272-273).

**Why was verbal informed consent used instead of written consent?**

Thank you. We recognized that people were not necessarily able to speak freely about government actions in all countries and wanted to protect our sources while verifying publicly-available information. In addition, we wanted to minimize burdens associated with participation such as not having access to software programs allowing signatures or by having to print and scan documents. We have added this explanation to the text (Clean manuscript lines 276-278).

**What is the plan for sharing the findings with the participants?**

Case reports are published publicly on the COVID-19 Policies and Epidemiology Website (<https://covid19-policies.healthsci.mcmaster.ca/research/publications/>).

**• Did you conduct the key informant interviews in-person or remotely? If remote, what technology was used? What was the language of the interviews?**

We conducted interviews in English, remotely using Zoom. We have clarified in the text (Clean manuscript lines 227-228).

**How was the data handled? How was transcription conducted?**

Audio recordings were produced using Zoom (version 5.9.3). SM created verbatim transcripts from recording. All data were stored securely on local devices that were password protected. We have clarified in the text (Clean manuscript lines 229-233).

**Results**
**• Did subnational levels of government have a say on the implementation strategy for the national mitigation/containment policies? Did that impact the outcome in some way? This will be good to know since you specifically excluded documents on subnational responses.**

Thank you. We have explained the decision-making structure in both countries on the national level in the results section of the paper. In South Africa, the National Coronavirus Command Council (NCCC) comprised only of members of the federal cabinet who made decisions on the COVID-19 policy response. In Uganda, there was a National Task Force (led by the President) who made policy decisions. We have added more specific language in the manuscript text to clarify (Clean manuscript lines 411 and 420).

**• Page 15 line 294: SA and Uganda are described as highly populated. What is this relative to?**

Thank you. We were intending to explain that the population sizes of South Africa and Uganda are comparable. We have updated the language in the manuscript text (Clean manuscript line 323).

**• Page 16-17: line 330-340: I am not sure of the relevance of the discussion on global health security ranking given how far off the index was for predicting COVID-19 outcomes in the ranked countries. Consider deleting to cut the word count.**

Thank you. Based on your comment on the length of the paper, we moved the Global Health Security (GHS) Index data to Table 2 to reduce the word count. We agree that the Global Health Security Index did not necessarily predict COVID-19 outcomes in South Africa and Uganda; however, since these indicators were selected a priori, it was not possible to know how far off the index was prior to completing this analysis. In retrospect, we felt it was worth using the GHS Index to highlight that indicators used to assess a country’s pandemic preparedness need to be re-evaluated in light of findings from COVID-19 to include factors which made country responses successful. We have added this information to the discussion section, under implications for research (Clean manuscript lines 855-860).

**• It may be insightful for the reader if per capita health expenditure in SA and Uganda are provided.**

Thank you. We used health expenditure as a percent of GDP as one of the indicators we collected a priori. This information has been added to Table 2 on contextual factors (Clean manuscript lines 311-312)**.**

**• Page 18 line 365: what is the date for the Uganda inequality score?**

Thank you. Uganda’s Gini index (inequality) score is from 2016. The year has been added to Table 2 (clean manuscript lines 311-312).

**• Page 19 line 387-388: linking HIV prevalence to immunocompromise may be incomplete without indicating the proportion of infected people who are on antiretroviral treatment.**

Thank you. We agree that HIV prevalence may not provide a complete understanding of immunocompromise in these countries and have removed this sentence. We were relying on global indicators to ensure comparability between contextual factors. We chose to use prevalence of HIV as a proxy indicator due to a lack of availability of other appropriate measures of immunocompromise. We added wording to explain in the text (Clean manuscript lines 194-197).

**Discussion**
**• There is an absence of a discussion on isolation policies for infected people. Were those not considered relevant for the epidemiology of COVID?**

Thank you. We agree that these policies are relevant to COVID-19 epidemiology. We have added a section describing quarantine for travellers, confirmed cases, and contacts to the results, and mentioned them in the discussion (Clean manuscript lines 569-576, line 716, and lines 734-735).

**Reviewer #3:**

**This is a well written paper which is providing important information that can inform future pandemic responses on the continent and globally. Therefore the chosen research topic is relavant. However individual country responses are also complex which may require more extensive and comprehensive research.**

Thank you! Yes, these country policy responses are nuanced and need to be understood within their contexts.

**South Africa and Uganda have different government systems, human rights profiles and freedoms. This informs responses to state led efforts. In contexts where there are authoriatrian elements enforcements and responses to restrictions may be limited due to suppression of freedoms. South African is also the 3rd largest economy on the continent and has some of the most advanced infrastructure and leading research institutions on the continent. This has implications on a comparative analysis. I would recommend the authors to include in the context of the two countries more information about health systems in both countries especially human resources , financing and public health infrastructure.**

Thank you. We recognize these factors are important for comparing COVID-19 policies between countries. This comment relates to those of Reviewer #1 and #2. As we mentioned, we began our research with a list of global indicators a priori. With regards to government systems and human rights profiles and freedoms, we collected the following indicators: Fragile States Index, Global Freedom scores, Internet Freedom scores, Trust in national government, Types of government (authoritarian vs democratic), Index of Economic Freedom and World Press Freedom Index. Data on these indicators can be found in the country-specific case reports and in our online database which are available at our website: <https://covid19-policies.healthsci.mcmaster.ca/>. However, these indicators were not the main focus of this paper.

Based on Reviewer #2’s comments, we have added the current health expenditure as a percentage of GDP to Table 2. To address your comment on health human resources, we’ve also added the physician density (physicians per 1000 population) to Table 2 and mentioned it in the text (Clean manuscript lines 311-312 and 344-346).

**With regards to epidemiological data, our world in data is a very useful resource however data quality and comprehensiveness highly depends on publicly available data sources. The available data therefore may differ from country to country depending on individual country information systems and public platforms with available data. The authors did acknowledge limitations of relying on publicly available data but i would encourage them to triangulate data from our world in data with information from the regular situation analysis released by respective ministries of health.**

Thank you. We agree that publicly available data have limitations. These limitations are discussed in detail by the COVID-19 Policies and Epidemiology Working Group in a separate paper. We have provided a citation in the text (Clean manuscript line 816).

**The authors did well in comparing factors that contribute to possible severity and progression of the diseases which could potentially explain the mortality rates and severe cases recorded.**

Thank you, there are many possible factors to consider!

**However when it comes to overal analysis of the impact of various policies on the progression of the pandemic its important to note that its complex and multifaceted influenced by various factors therefore a more in depth individual country case studies would have yielded more information that will be more useful for multi-country analysis.**

Thank you. We conducted individual, in-depth case studies for each country to inform this comparative analysis. The individual reports are cited and are available at our website (<https://covid19-policies.healthsci.mcmaster.ca/research/publications/>). The citations and link are included in the methods section of the manuscript (Clean manuscript lines 264-265).
